# Destabilization damage characteristics and infrared radiation response of coal-rock complexes

**DOI:** 10.1038/s41598-024-65029-w

**Published:** 2024-06-18

**Authors:** Bo Li, Zhen Shi, Mengsheng Jiang, Junxiang Zhang, Li Li

**Affiliations:** 1 College of Safety Science and Engineering, Henan Polytechnic University, Jiaozuo, 454003 China; 2 State Key Laboratory Cultivation Base for Gas Geology and Gas Control, Henan Polytechnic University, Jiaozuo, 454003 Henan China; 3 State Collaborative Innovation Center of Coal Work Safety and Clean-Efficiency Utilization, Henan Polytechnic University, Jiaozuo, 454003 Henan Province China; 4 College of Safety Science and Engineering, Xinjiang Institute of Engineering, Urumqi, 830023 China; 5 School of Energy and Environment Engineering, Zhongyuan University of Technology, Zhengzhou, 450007 China; 6 Ministry of Education, Key Laboratory of Safety and High-Efficiency Coal Mining, Anhui University of Science and Technology, Huainan, 232001 Anhui China

**Keywords:** Coal-rock complexes, Damage patterns, Infrared thermal image differentiation, Stress field evolution, Damage evolution, Coal, Petrology

## Abstract

To investigate the characteristics of destabilization damage in coal-rock complexes. Mechanical property tests were conducted on coal, rock, and their complexes. An infrared thermal camera was employed to real-time monitor the infrared (IR) radiation response signals during the destabilization damage process. A numerical model of coal-rock destabilization damage was developed, and its validity was verified. Deformed stress fields and displacement contours were obtained during the destabilization damage process. Upon destabilization, numerous cracks form at the base of the “coal” section, extending towards the interface, resulting in the formation of a wave-like deformation region. The differentiation in infrared thermal images is more pronounced in the “coal” section compared to the “rock” section. A high-stress region is evident at the interface, resulting in an area of high stress differentials. However, the bottom of the “coal” section also exhibits a region with high stress differentials and a more pronounced tendency towards destabilization damage. Displacement contours revealed that numerous units at the bottom of the “coal” section had slipped and misaligned, leading to the accumulation of damage and an elevation in the local damage level. It is a crucial factor contributing to the notable phenomenon of IR thermal image differentiation.

## Introduction

Given that coal resources are a crucial part of China’s energy composition, safe and efficient mining technology has become extremely important. The depth of coal mining has been growing year by year under the circumstances of gradually depleted shallow coal resources for the purpose of meeting the demand for energy production and consumption. At present and for a period of time in the future, the technology for coal mining in the depth remains the research focus of relevant scholars^1–3^. The complicated environmental factors (such as high temperature and high pressure), the differences in geological properties and the morphological structure in deep mines jointly lead to the formation of a complex geological environment^4–8^. Coal mining work, be it excavation, mining or the surrounding rock bearing system of a roadway, inevitably involves the load-damage problem of complexes. Due to the disparity in coal and rock strength, there is a discontinuous transfer of force and energy within the geological formation. This occurs due to the combined impact of the original rock stress and mining-induced stress, leading to localized energy accumulation within the formation. This, in turn, instigates a significant dynamic instability phenomenon. Such a phenomenon has a profound impact on the safe production of coal mines, particularly in the context of mining operations in deep coal mines under high geostress conditions. Therefore, studying the load-damage process of the complexes and exploring their damage characteristics and corresponding damage rules are crucial for the safe and efficient mining work in deep coal mines.

The load-damage of coal rock has been broadly researched on by scholars all over the world^9^. They often define the damage criteria form the perspectives of stress and load work and generally believe that the stress–strain of coal rock is bound up with the accumulation and dissipation of energy^10–15^. However, some scholars hold the view that the load-damage of coal rock is an energy-driven instability phenomenon, and regard energy accumulation as the essential feature of rock deformation and damage^16,17^. From the point of view of energy, Luo et al.^18^ studied the dynamic stress balance of sandstone under impact loading and found that the dynamic strength of sandstone is closely related to energy dissipation. Razavi et al.^19^ adopted the average strain energy density criterion to predict the crack load of rock samples, which is in good consistency with the crack load obtained from the experiment. Liu et al.^20^ derived crack locations and energy accumulation before and after the appearance of cracks in the critical strata, and simulated the critical energy value of rock burst induced by cracks according to the deformation rate and energy release of surrounding rocks. Since the conversion between load work and strain energy is the intrinsic cause for coal rock damage, it is vital to explore the energy conversion features of coal rock strain damage under varying loading conditions in the mining work^21–24^. The study from the aspect of energy conversion is conducive to effectively explaining the strain damage rules of coal-rock complexes, analyzing their load-damage characteristics in depth and expounding the conversion mechanism of load work-strain energy. By monitoring the IR thermal image differentiation characteristics during the evolution of the temperature field of the coal-rock, the IR imager can study the destabilization damage characteristics and damage evolution mechanism of the coal-rock from the perspective of frictional heat effect. Recently, numerous researchers have reported the load-damage of coal-rock complexes and performed loading, impact and cyclic loading experiments considering factors such as strength ratio and height ratio. Some conclusions are listed as follows. Song et al.^25^ conducted cyclic loading experiments with different coal-rock combination forms and found that the sample damage mainly exhibits three modes, i.e., shear slipping, tensile splitting and cracking. The results of uniaxial compression experiments by Li et al.^26^ led to the conclusion that progressive damage of the “coal” section will induce rebound deformation of the “rock” section. Zheng et al.^27^ investigated the effects of factors such as effective coal-rock height ratio, interface angle and rock thickness on the mechanical properties of coal-rock complexes, and found that effect of effective coal-rock height is greater than that of coal strength. Liu et al.^28^ investigated the damage characteristics of coal-rock complexes with rough and discontinuous interfaces, revealing that the composite structure of coal-rock complexes becomes more stable gradually as the roughness increases. Xue et al.^29^ explored the crack characteristics and instability of coal-rock interlayer. It is concluded that the higher the ratio of rock, the lower the damage degree of the sample after unloading. In their work on the brittle damage characteristics of coal-rock complexes under uniaxial loading, Gao et al.^30^ disclosed that the brittle damage belongs to sliding and tensile cracking induced by frictional resistance. Liu et al.^31^ conducted relevant experiments with regard to the damage characteristics of coal in coal-rock complexes, and further established a coal damage constitutive model in which the damage body and the Newtonian body were connected. Lu et al.^32^ proposed a multi-scale fusion module endowed with spatial features and dynamic differential features, which in turn realized the prediction of coal-rock crack evolution. Chen et al.^33^ simulated the influence of loading rate on the stress–strain behavior of coal-rock complexes. The results indicated that the loading rate will increase the width of the main shear damage zone. Numerous studies have been undertaken through experiments, engineering analyses, and numerical simulations. These investigations primarily concentrate on elucidating the impacts of factors such as height ratio, interface roughness, interface dip angle, and the strength of coal-rock on the comprehensive mechanical properties and damage characteristics of coal-rock complexes^34–38^. Currently, various studies have investigated the infrared radiation response characteristics during the destabilization process of rock bodies. However, the destabilization process of coal-rock complex exhibits a discontinuous stress transfer, making it more prone to local damage escalation. Additionally, the phenomenon of infrared thermal image differentiation becomes more pronounced. This study aims to examine the infrared radiation response characteristics of the complexes destabilization process. Such an investigation is crucial not only for unveiling the damage evolution mechanism in the destabilization process of the complexes but also for providing a reference for predicting destabilization in complexes. To achieve this objective, the study focuses on coal, rock monomer, and their complex’s destabilization damage morphology and infrared thermal image differentiation characteristics. The analysis delves into the temperature field evolution behavior during the process of complexes destabilization damage, elucidating the intrinsic mechanism of the infrared thermal image differentiation phenomenon in the destabilization process of the complex. This research holds significant implications for the safe mining of mines.

In this study, an infrared imager is employed to real-time monitor the process of destabilization damage in coal, rock, and their complexes. The study compares stress–strain behaviors and destabilization damage patterns while analyzing the evolution of destabilization damage behavior in the complexes through infrared thermal image differentiation. The numerical model for destabilization damage in coal-rock is constructed based on the generalized Mises yield and damage criterion, along with the Drucker–Prager (D–P) modified model^39^. The validity of the model is verified by comparing the damage pattern of the sample with the equivalent plastic deformation contours of the model. The analysis of the expansion pattern of cracks on the surface and interior of the sample during destabilization is conducted using equivalent plastic deformation contours. Additionally, the distinctive evolution behavior of the stress field during the destabilization process of the complex is discussed. The study investigates the damage evolution in the process of destabilization and damage in the complexes by combining displacement contours and IR radiation response characteristics.

## Experimental preparation and load-damage numerical model

### Experimental preparation and scheme

**Figure 1:**
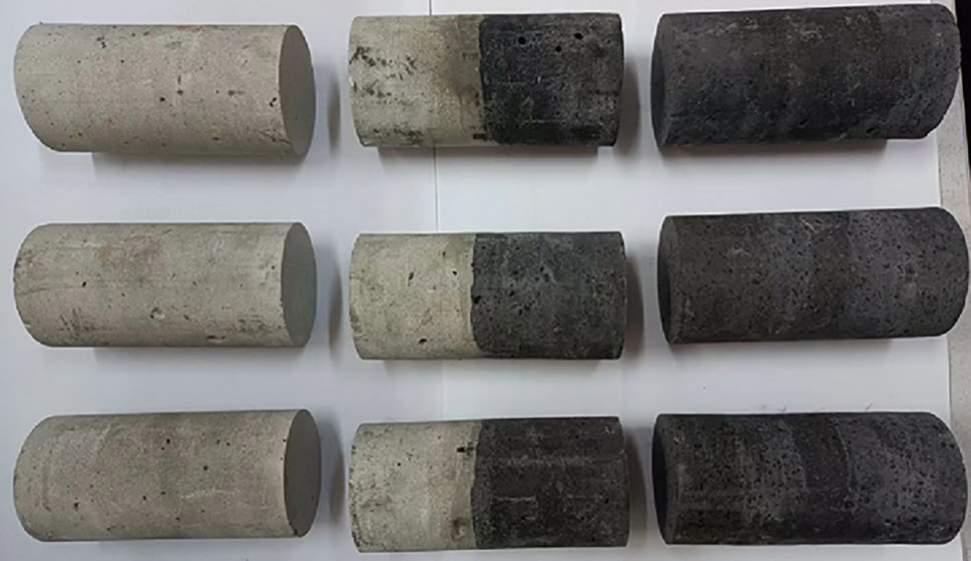
Processed samples.

**Figure 2:**
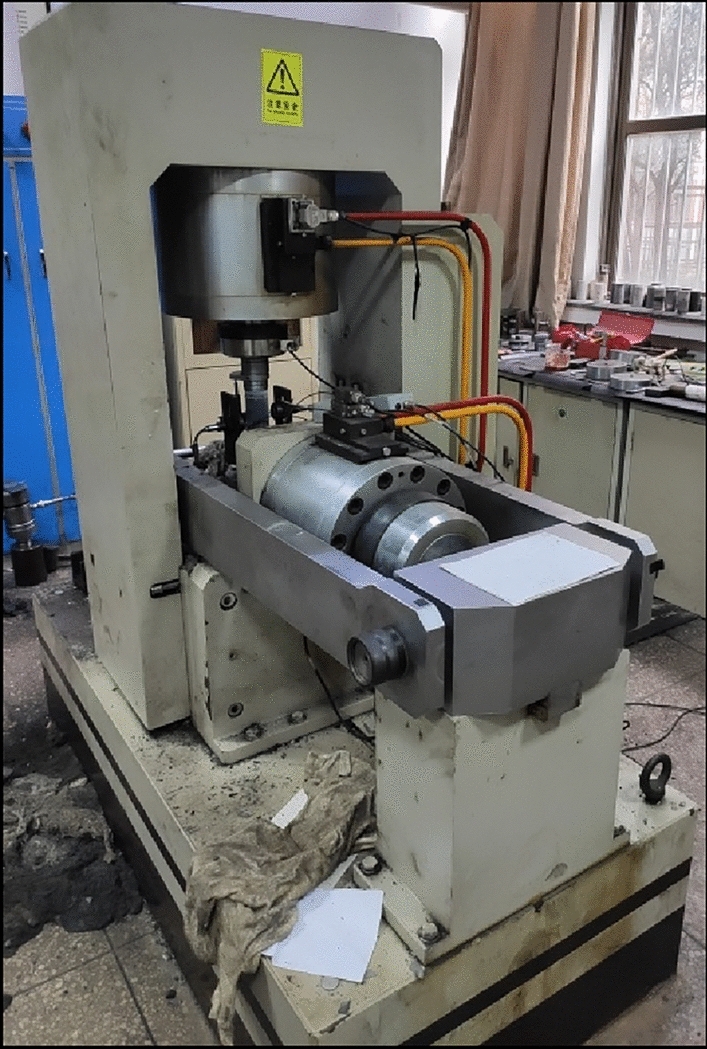
Mechanical property test system.

For the purpose of conducting experiments on the load-damage rules of coal, rock, and their complexes, samples were fabricated using a similar material casting method^40^. The “rock” section comprised silicate cement, river sand (particle size ≤ 3.5 mm), and water in a mass ratio of 1:1:0.5, while the “coal” section consisted of coal dust (particle size ≤ 5 mm), silicate cement, and water with a mass ratio of 1:1:0.45. Under constant conditions of 20 ℃ temperature and a relative humidity not less than 95%, the curing process for the cast specimens extended over 28 days. Standard cylinders measuring Φ50 × 100 mm were drilled and chosen as representative samples for rock, coal, and their complexes. Figure 1 illustrates the prepared samples, and Table 1 provides details regarding sample grouping. The mechanical property test system, as depicted in Fig. 2, was a digitally controlled electro-hydraulic servo RMT-150 tester that utilized travel control and operated at a loading rate of 0.5 mm/s. The specific parameters of the test system are presented in Table 2. All samples underwent uniaxial load-damage experiments using the mechanical property test system to derive their stress–strain curves and damage characteristics. The Fotric225s thermal imager monitored temperature trends in real time during the loading of samples.

**Table 1:** Information of sample grouping.

Samples No	C-1	C-2	C-3	R-1	R-2	R-3	CR-1	CR-2	CR-3
Weight (g)	286.1	286.1	286.1	286.1	286.1	286.1	286.1	286.1	286.1
Height (mm)	100.1	100.2	99.9	99.7	100.1	100.2	100.1	99.8	100.2
Diameter (mm)	49.8	49.9	50.2	49.8	50	50.2	49.8	50.1	49.9
Densities(kg/m^3^)	1467.4	1457.5	1458.6	2012.4	1973.6	1979.1	1738.7	1751.5	1751.9
Average densities (kg/m^3^)	1461.14			1988.367			1747.38

**Table 2:** Experimental parameters of the test system.

	Maximum load	Maximum confining pressure	Deformation rate	Loading rate
Mechanical property test system	1000 KN	50.0 MPa	0.0001–1.0 mm/s (Level 13)	0.01–100.0 KN/s (Level 13)

### Destabilization damage numerical model

**Figure 3:**
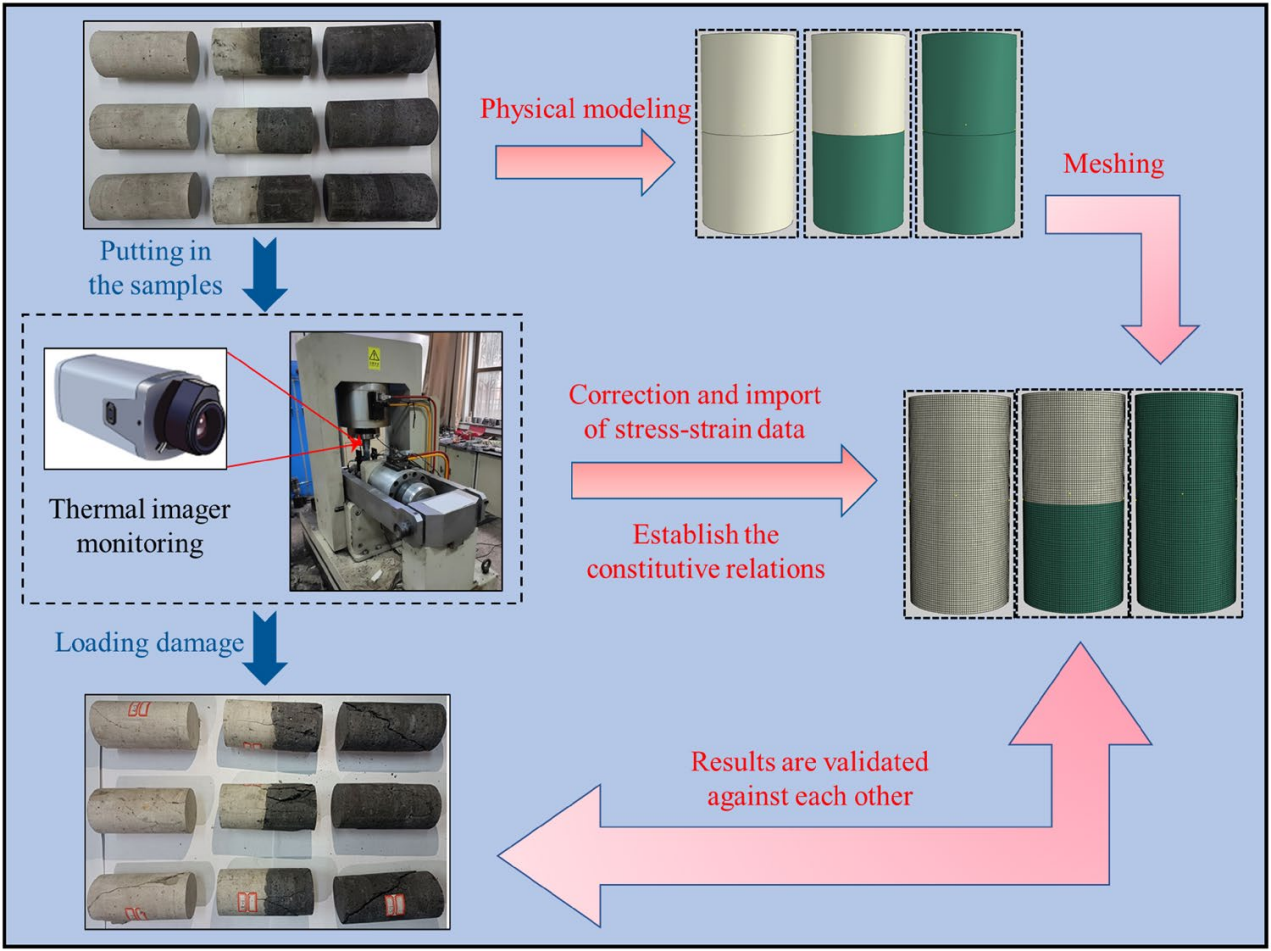
Model establishment.

The fundamental parameters of the numerical model were adjusted by integrating the damage modes observed in physical experiments with the simulation results of stress field evolution. Subsequently, the research focused on the evolution law of the stress field in the complexes during the destabilization damage process. Simultaneously, the study investigates the characteristics of destabilization and damage patterns in coal-rock complexes through a comparative analysis of IR radiation response characteristics and the evolution process of displacement contours calculated by the model. Scale physical modeling was conducted based on the physical experimental dimensions of Φ50 × 100 mm, and partitions were established to assign properties to different areas of the model. The entire model was partitioned into 242,000 C3D8R units (8-node hexahedral linear reduced integral units), as depicted in Fig. 3.

The damage behavior of coal rock during the load-compression process comprises both brittle and ductile damage. For a more accurate simulation of the material damage process, the stress–strain behavior of the model material adheres to an elastic–plastic constitutive relation. Utilizing the D–P criterion, the modified and simplified stress–strain experimental data from coal-rock samples were incorporated as the hardening rule of the model to achieve the simulation of elastic–plastic damage in coal rock^41^. The load-induced damage in coal rock can be considered as the synergistic effect of principal stress and corresponding partial stress. This effect is more accurately represented by adopting the modified D–P criterion. The model employs the maximum relative displacement of the unit as the criterion for assessing the failure behavior of the unit. Subsequently, it simulates specimen cracking by subjecting a portion of the unit to continuous strain until failure occurs. Eq. (1) represents the functional expression of the linear D–P model. Here, $t = q$ when $K = 1$. The yield surface conforms to the Mises yield criterion with regard to a circular yield surface^42–44^.

1 $$F = t - p\tan {\upbeta - }d$$

2 $$t = \frac{1}{2}q\left[ {1 + \frac{1}{K} - \left( {1 - \frac{1}{K}} \right)\left( \frac{r}{q} \right)^{3} } \right]$$

where $F$ is the elastic–plastic damage yield function; $t$ is another form of partial stress, $p$ is the principal stress; $d$ is another form of internal cohesion; $q$ is the partial stress; $K$ is the tensile strength-compressive strength ratio; $\upbeta$ is the friction angle of yield surface on the $p$-$t$ stress surface.

**Figure 4:**
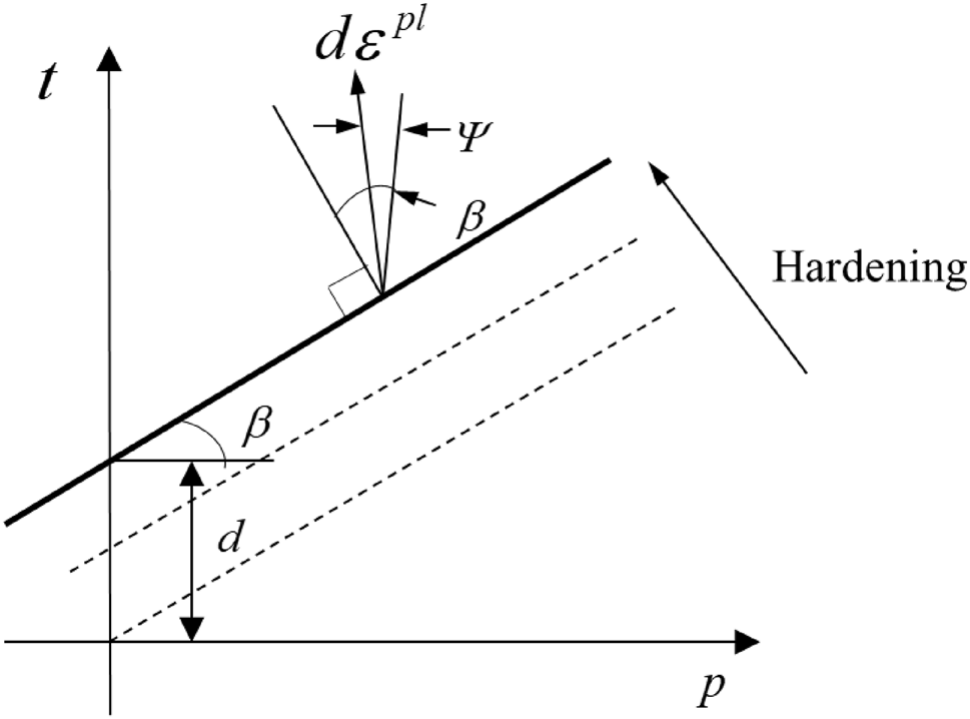
Linear D–P model: yield surface and flow direction in the $p$–$t$ stress plane.

In contrast to the yield surface, the flow rule governing the plastic potential surface of the linear D–P model is non-associative, following Eq. (3). The depiction of the linear D–P model is presented in Fig. 4 ($d\varepsilon^{pl}$ is the equivalent plastic strain.)^45^.

3 $$G = t - p\tan \Psi$$

where $G$ is the plastic potential function; and $\Psi$ is the expansion angle on the $p$–$t$ stress surface.

For enhanced comparison and analysis of destabilization damage pattern characteristics in physical experiments of coal rock samples, the nominal stress–strain is converted into real stress–strain data in the model, and an isomorphic model based on the D-P criterion is constructed. Additional basic parameters of the model are detailed in Table 3.

**Table 3:** Basic physical parameters of the model.

	Rock	Coal
Density (kg/m^3^)	1988	1461
Friction angles (^o^)	60	64
Dilation angles (^o^)	2	10
Elastic modulus (GPa)	11.03	3.36

The plastic strain increment in the linear D–P criterion can be defined by Eqs. (4), (5), (6).

4 $$d\varepsilon^{pl} = d\overline{\varepsilon }^{pl} \frac{1}{{\left( {1 - \frac{1}{3}\tan \psi } \right)}}\left( {\frac{\partial q}{{\partial {\varvec{\sigma}}}} - \tan \psi \frac{\partial p}{{\partial {\varvec{\sigma}}}}} \right)$$

5 $$d\varepsilon^{pl} = d\overline{\varepsilon }^{pl} \frac{1}{{\left( {1 - \frac{1}{3}\tan \psi } \right)}}\frac{\partial }{{\partial {\varvec{\sigma}}}}\left( {t - p\tan \psi } \right)$$

6 $${\varvec{\sigma}} = (\sigma_{1} ,\sigma_{2} ,\sigma_{3} )$$

Assuming that the plane strain is in the direction of the maximum principal stress $\sigma_{1}$, the principal stresses $\sigma_{1}$, $\sigma_{2}$ and $\sigma_{3}$ can be rewritten in accordance with the plastic plane principal stress $p$ and the partial stress $q$. Eqs. (7), (8), (9) show the conversion relations.

7 $$\sigma_{1} = \frac{1}{2}(\sigma_{2} + \sigma_{3} ) - \frac{1}{3}q\tan \psi$$

8 $$q = \frac{3\sqrt 3 }{{2\sqrt {9 - \tan^{2} \psi } }}(\sigma_{2} - \sigma_{3} )$$

9 $$p = \frac{\tan \psi }{{2\sqrt {3\left( {9 - \tan^{2} \psi } \right)} }}(\sigma_{2} - \sigma_{3} ) - \frac{1}{2}(\sigma_{2} + \sigma_{3} )$$

By combining Eqs. (1), (8) (9), a linear D–P criterion yield surface function expressed by principal stresses $\sigma_{2}$ and $\sigma_{3}$ can be obtained.

10 $$F = \frac{9 - \tan \psi \tan \beta }{{2\sqrt {3\left( {9 - \tan^{2} \psi } \right)} }}(\sigma_{2} - \sigma_{3} ) + \frac{1}{2}\tan \beta (\sigma_{2} + \sigma_{3} ) - d$$

Combining Eqs. (1), (4), (5), (10), the constitutive relation of coal-rock complexes undergoing stress–strain is thoroughly explained. Based on the segmentation of coal and rock partitions, the C3D8R unit properties grid is chosen. The elastic–plastic constitutive relationship of the unit is established using the parameters in Table 3, experimental stress–strain data, and Eqs. (4), (5), (6), (7), (8), (9), (10). The judgment of the unit’s failure behavior relies on the maximum relative displacement. This approach facilitates the simulation and resolution of cracking behavior, leading to the development of a finite-element numerical model for destabilization damage in coal-rock complexes. The model is solved using the ABAQUS/Explicit solver.

## Analysis of results

### Strength characteristics and destabilization damage patterns

**Figure 5:**
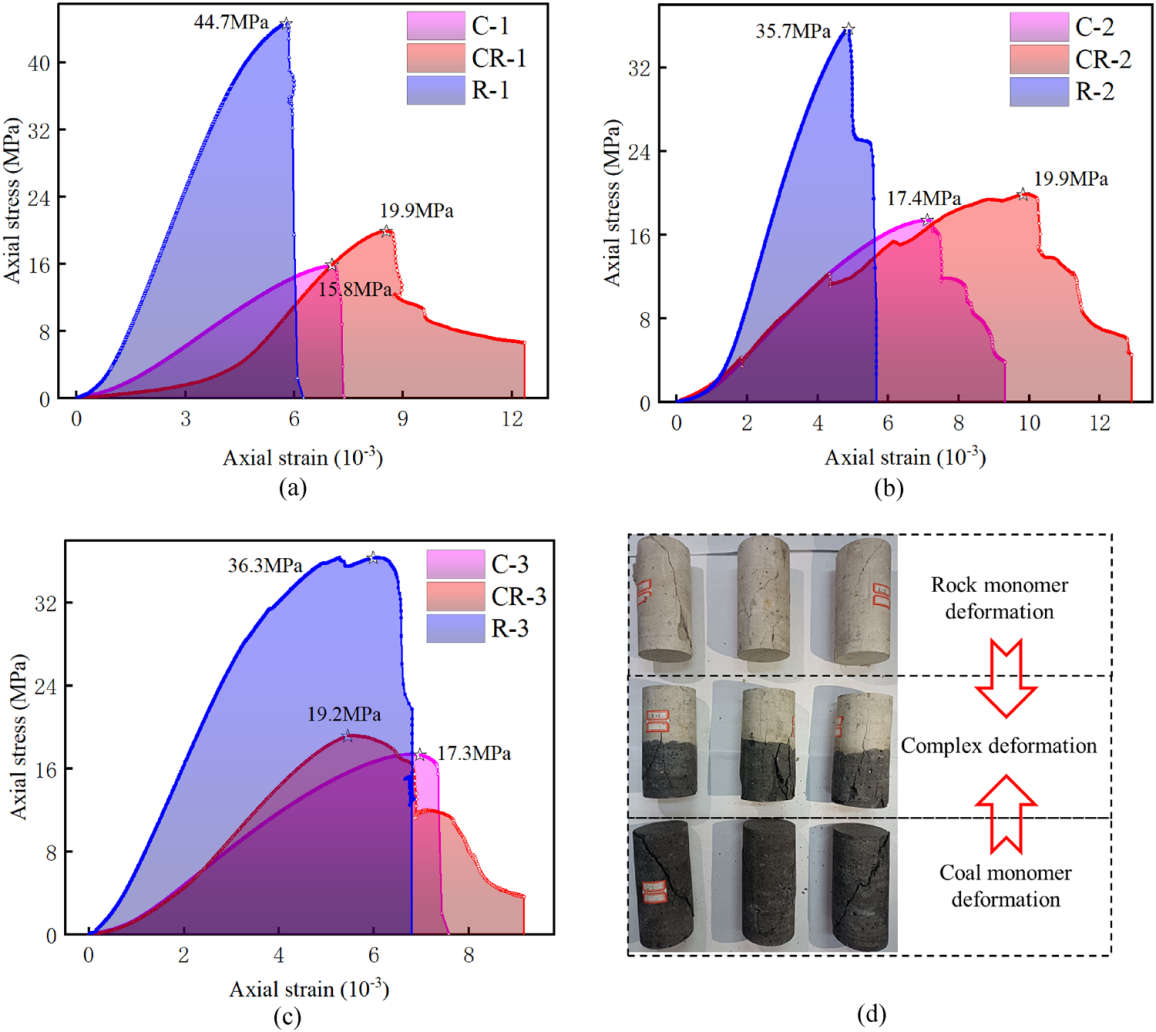
Stress–strain curves and load-damage characteristics. (**a**) Curves of group 1. (**b**) Curves of group 2. (**c**) Curves of group 3. (**d**) Comparison before and after samples are subject to load-damage.

The substantial variation in mechanical properties among different areas within the coal-rock complexes leads to the following phenomenon. During the loading process, the high-strength area, while being “loaded,” also acts as the carrier of the “load”. In contrast, the low-strength area becomes the focal point of damage, serving as the primary region where strain energy accumulates. Figure 5 illustrates the stress–strain curves of coal, rock, and their complexes obtained in the mechanical property experiments. The maximum uniaxial compressive strength (UCS) exhibits a trend of R > CR > C.

**Figure 6:**
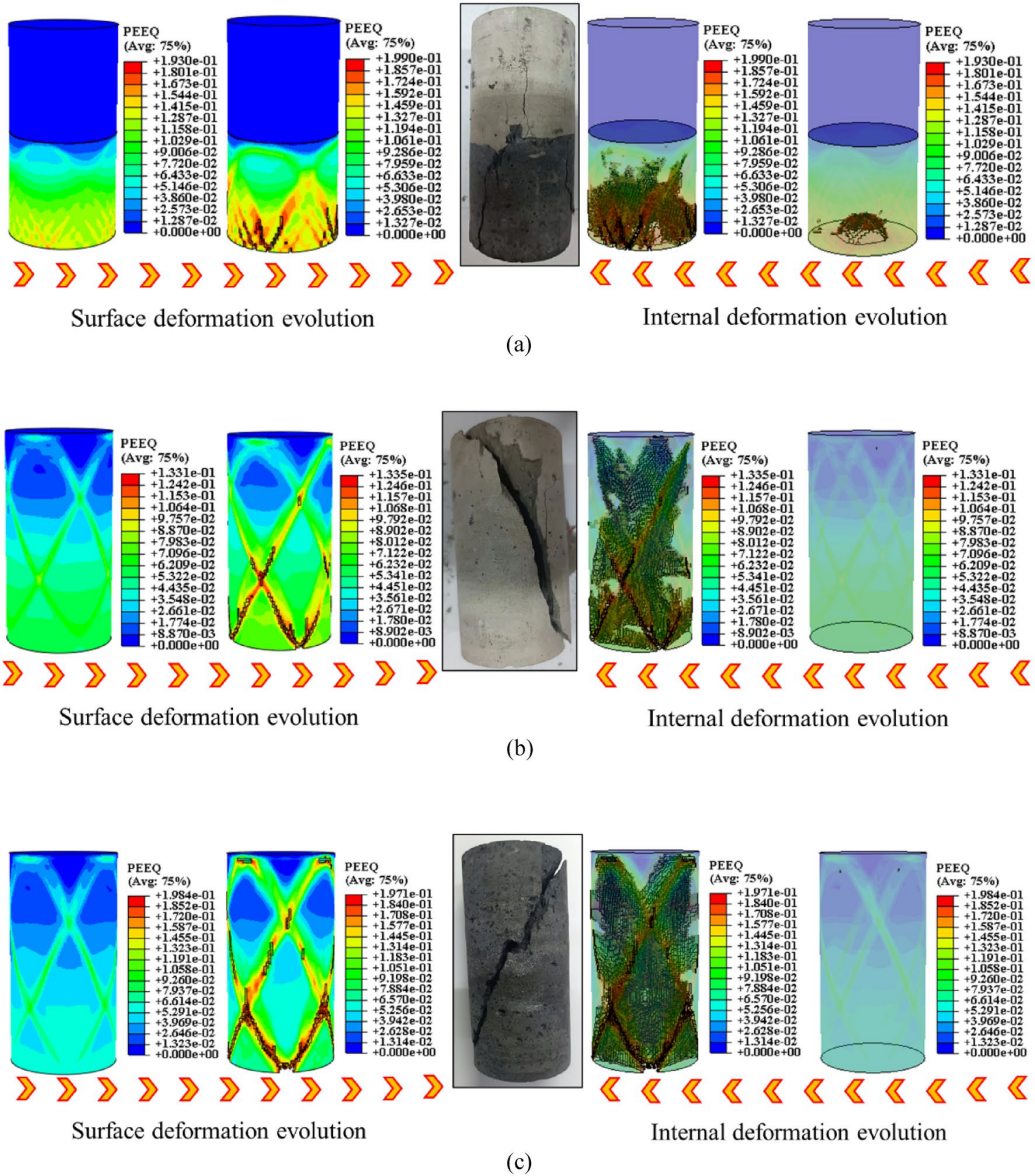
Characteristics of destabilizing damage patterns of coal and rock monoliths and their complexes. (a) Complexes. (b) Rock. (c) Coal.

As illustrated in Fig. 5, under the condition of “strong rock and weak coal,” the UCS of the complexes falls between that of coal and rock. It experiences an increase of 11–25.9% compared to coal and a decline of 44.3–54.3% compared to rock. During the experiment, the UCS of the “coal” section governs the damage in the coal-rock complexes. However, the overall UCS of the coal-rock complexes surpasses that of the “coal” section. This phenomenon can be explained from two perspectives. Firstly, the destruction of the “coal” section increases. Secondly, the “rock” section of the structure absorbs some loads and deforms. Additionally, Fig. 5a–c illustrates that when the axial stress decreases dramatically, the axial strain of the final specimen exhibits a magnitude relationship of CR > C > R. This suggests that the overall degree of destabilization damage in the complexes is more severe. For a thorough analysis of the destabilization damage pattern in coal, rock, and their complexes, the equivalent plastic strain pattern of the coal-rock destabilization damage model was calculated. The results of the destabilization damage pattern of samples are highly consistent with the equivalent plastic strain pattern of the model. As shown in Fig. 6 (unit: mm).

In Fig. 6a, when the elastic modulus ER of the coal-rock complexes is approximately three times as high as that of coal (i.e. ${\text{E}}_{{\text{R}}} \approx 3{\text{E}}_{{\text{C}}}$), the “coal” section undergoes plastic deformation, and the size and extent of plastic deformation decrease as the interface is approached. During the loading process, the high-strength “rock” primarily acts to transfer the load work, and the combination of “coal” and “rock” at the interface enhances the deformation resistance of “coal” in that vicinity. The reason for this lies in the discontinuity of stress transfer within the complex. The inherent strength of the “rock” component enables it to transfer stresses more efficiently. Additionally, coal and rock represent distinct areas of the same geometry, implying that the interface bonds the coal-rock complex with higher strength. A comparative analysis of the damage patterns of coal, rock, and their complexes in Fig. 6 is conducted through color classification of deformation degree classes. In the early stages, the coal/rock samples exhibit green bands in an “X” shape, with the lower section displaying an overall tendency of green. Subsequently, reddish-yellow bands form, signifying the generation of macro-cracks. As the cracks expand, the samples will exhibit large “X”-shaped cracks. In the early stage of complex destabilization, the “coal” exhibits wavy cyan stripes, green stripes, and “X”-shaped reddish-yellow stripes from the interface to the lower end. Subsequently, macro-cracks originate from the lower end, leading to their expansion. The damage pattern of the “rock” part mirrors that of the test specimen, appearing blue and nearly strain-free. This outcome correlates with consistent friction angle and expansion angle in the monomer region, aligning with the model of an elastic–plastic ontological relationship. The unique destabilization damage pattern results from differences in the physical properties of coal and rock within the complex. Ultimately, the complex develops numerous intersecting “X”-shaped cracks at the lower end of the coal, extending towards the interface, and forms a deformation region with wavy cyan-colored bands at the interface. In the monomer, a few “X”-shaped cracks are generated in the middle and lower parts, crossing each other near the top and bottom ends. Greenish stripes, representing the tendency of crack expansion, form slant lines with a single expansion pattern. The physical differences between coal and rock induce stress transfer discontinuity, resulting in distinct destabilizing destructive behaviors of the monomer and the complex. This discrepancy is the primary cause behind the formation of the concentrated destructive location and heightened destructive degree in the destabilizing destructive morphology of the complex specimen during testing.

### IR thermal image differentiation characteristics

**Figure 7:**
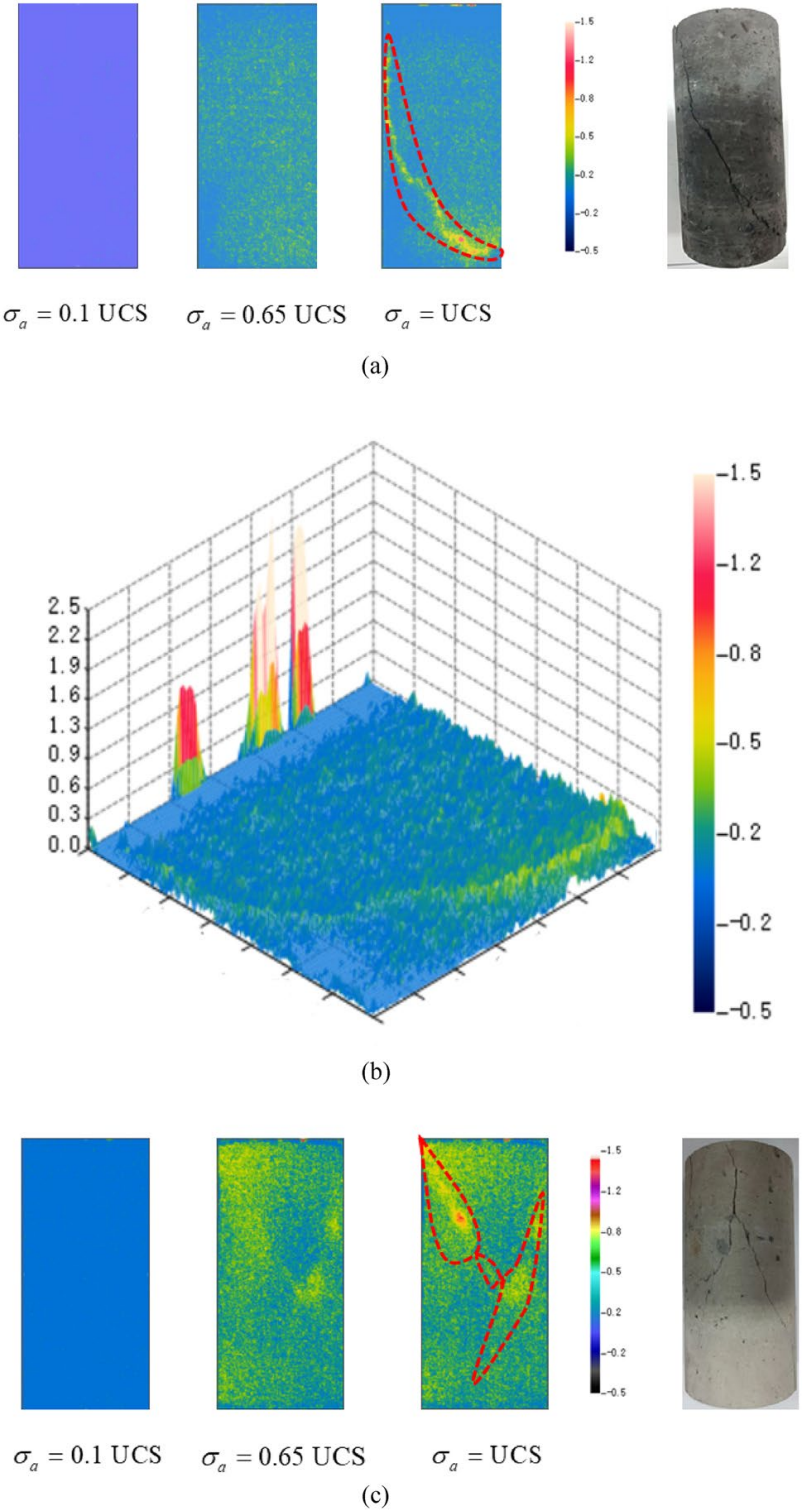

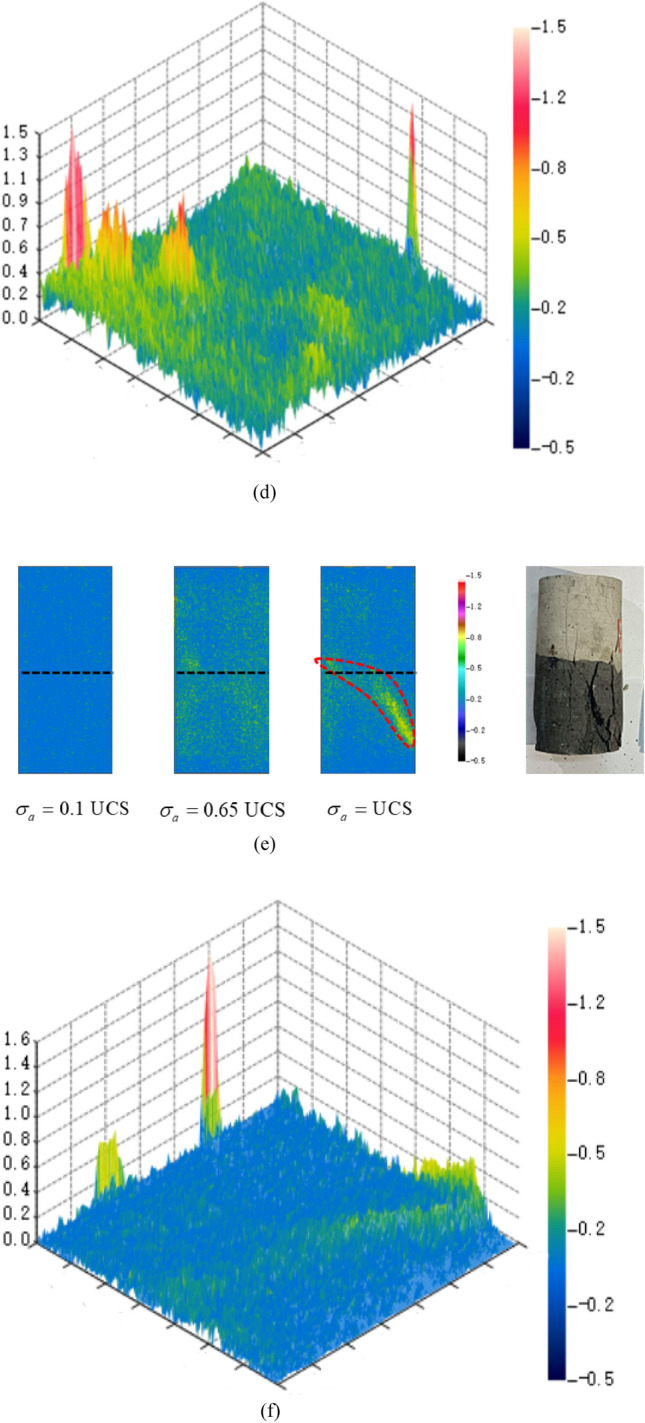
Infrared thermal images of the sample. (**a**) C-1. (**b**) 3D spatial temperature difference distribution (C-1: $\sigma_{a} = {\text{UCS}}$). (**c**) CR-1. (**d**) 3D spatial temperature difference distribution (R-1: $\sigma_{a} = {\text{UCS}}$). (**e**) CR-1. (**f**) 3D spatial temperature difference distribution (CR-1: $\sigma_{a} = {\text{UCS}}$).

The distribution and migration of the thermal field on the surface of the samples during the destabilization of coal-rock bodies are recorded by the infrared camera. This analysis discerns the differentiation and evolution characteristics to predict the location of destabilization damage by real-time monitoring of the IR temperature field on the samples’ surface. For the comparison and analysis of the infrared thermal image differentiation characteristics of coal, rock, and their complexes, the IR temperature field at three moments of axial loading stress $\sigma_{a} = 0.1{\text{UCS}}$, $\sigma_{a} = 0.65{\text{UCS}}$ and $\sigma_{a} = {\text{UCS}}$ is selected, and the amplitude of temperature difference is set to − 0.5–1.5 ℃, and the 3D spatial distribution chart of temperature difference values is plotted at the same time, as shown in Fig. 7 (unit: ℃).

In Fig. 7, the complex at $\sigma_{a} = 0.1{\text{UCS}}$ exhibits more pronounced differentiation characteristics compared to the monomer. This is attributed to the differing strengths of coal and rock, leading to discontinuous stress transfer within the complex and an increased likelihood of shear damage formation. In Fig. 7a, the C-1 surface at $\sigma_{a} = 0.65{\text{UCS}}$ exhibits a scattered distribution of yellow-green high-temperature areas, almost uniformly distributed. This indicates many small shear damages on the surface of the sample. When $\sigma_{a} = {\text{UCS}}$, the sample surface shows reddish–yellow high-temperature bands, and the infrared thermal image differentiation characteristics of the sample surface can be observed more clearly from Fig. 7b. At the upper end of the sample, a localized red high-temperature region appears. Simultaneously, a high-temperature region emerges at the location of the reddish-yellow stripe shown in Fig. 7a. The sample undergoes single-bevel shear damage along the high-temperature region, resulting in a macrocrack consistent with the destabilized damage pattern of the sample. In Fig. 7c at $\sigma_{a} = 0.65{\text{UCS}}$, R-1 exhibits a denser yellow–green scattering region than C-1. This indicates that the surface temperature increase is more significant when R-1 shows a destabilization damage trend. At $\sigma_{a} = {\text{UCS}}$, the surface of R-1 exhibits clear thermal differentiation on the infrared image, revealing a “Y”-shaped crossed reddish-yellow high-temperature bands. This pattern is consistent with the cracking pattern of the sample. Figure 7e, f reveal that the infrared thermal image differentiation and evolutionary features of CR-1 differ significantly from C-1 and R-1. At $\sigma_{a} = 0.65{\text{UCS}}$, the yellowish-green particles in the “coal” section are significantly larger and denser than in the “rock” section. Yellowish-green spots are also present at the coal-rock interface, representing the high-temperature region. At $\sigma_{a} = {\text{UCS}}$, the yellow-green stripes in CR-1 are monoclinic in the “coal” section, paralleling the interface, and remain scattered in the “rock” section. During the process of axial stress loading, micro-deformations occurred in the “rock” section, but no macroscopic cracks formed, and only the tendency to destabilization damage was observed.

According to the above analysis, the surface temperature field distributions of samples R-1 and C-1 are very similar, but the differentiation feature of R-1 is more pronounced. In CR-1, the “coal” exhibits more pronounced differentiation characteristics than the “rock”. Leveraging this feature, infrared thermography can be employed to monitor the infrared radiation temperature field of the surrounding rock. This facilitates the effective prediction of instability damage, particularly the more significant local radiation temperature divergence characteristics of coal-rock complexes, which proves highly beneficial for predicting instability damage. Such insights are of reference value for the safe mining of coal-rock complexes in the coal mining industry. The infrared thermal image differentiation characteristics of CR-1 align with the test and simulation results. The “rock” section exhibits minimal infrared differentiation and small amounts of micro-deformation, which are beneficial for increasing the UCS of the overall complexes. The “coal” and interface sections preferentially show destabilization damage tendency, produce macro-cracks, and increase the degree of destabilization damage. This is the main part of destabilization damage of coal-rock complexes.

## Discussion

### Stress field evolution

**Figure 8:**
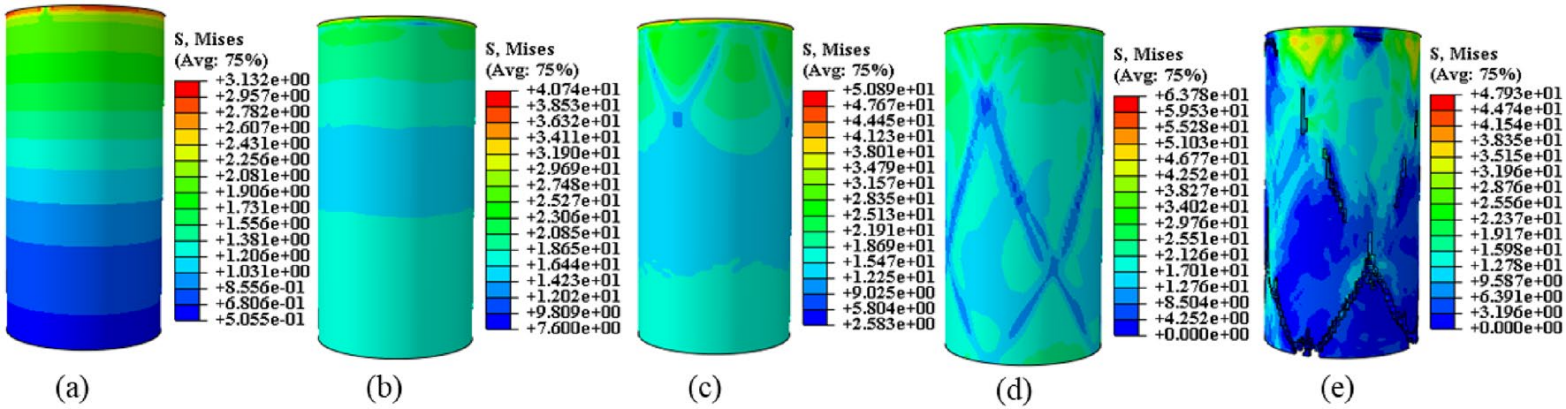
Coal monomer stress field evolution.

**Figure 9:**
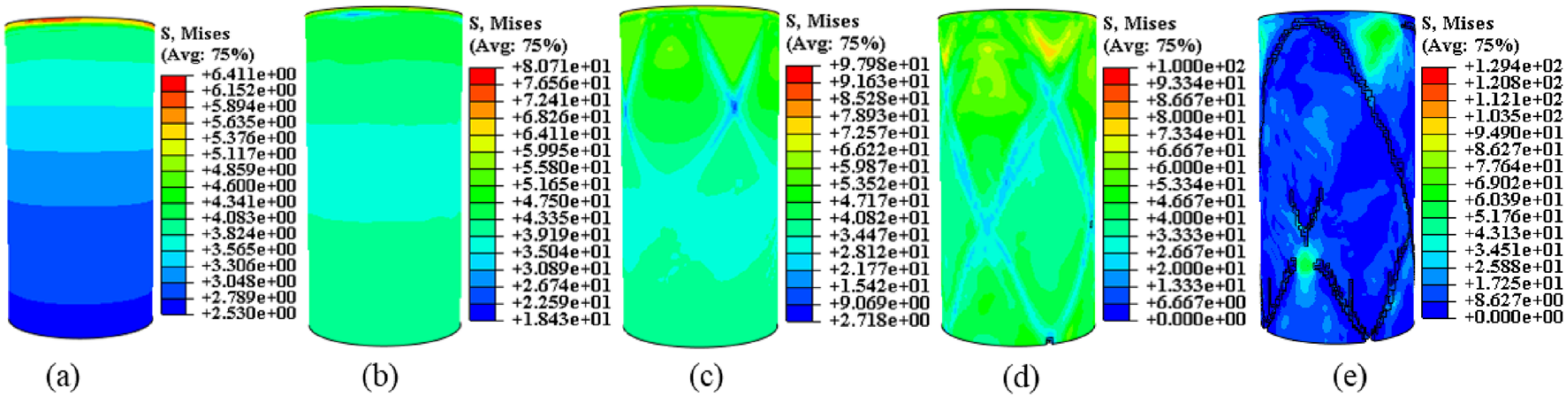
Rock monomer stress field evolution.

**Figure 10:**
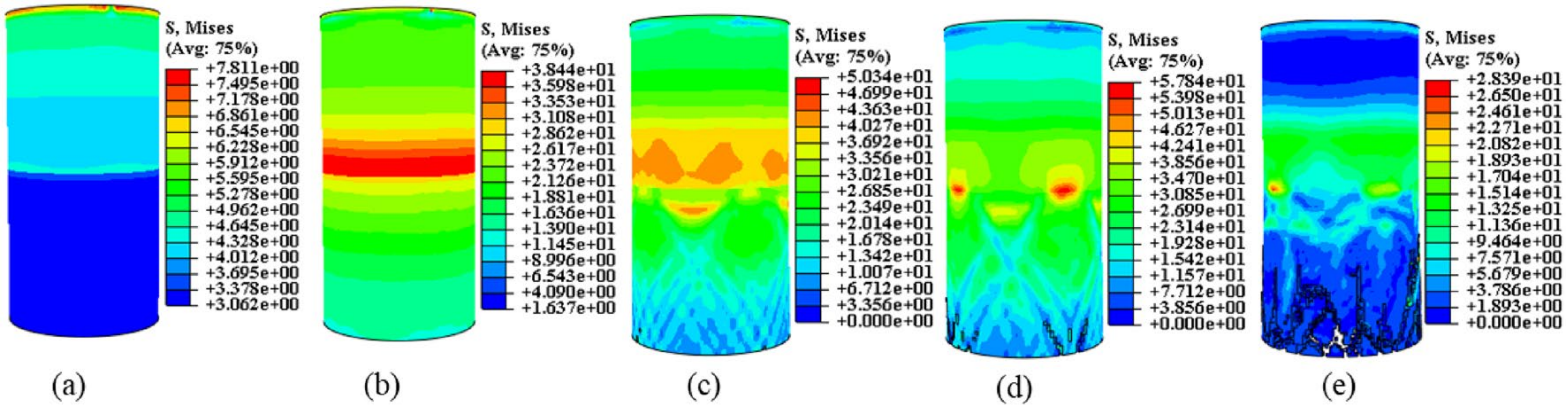
Complex stress field evolution.

After analyzing the strength characteristics and destabilization damage patterns of coal, rock, and their complexes, it was observed that the strength of the complex is more akin to coal than rock. Additionally, the degree of destabilization damage is more pronounced, with macro cracks predominantly concentrated in the “coal” section. During the destabilization damage process, infrared thermal image differentiation of the “rock” section of the complex shows a large number of shear-type micro-deformations. To better study the destabilization damage and damage evolution in coal-rock complexes, the analysis is conducted considering the behavior of stress field evolution. The stress contours resulting from the post-processing of the numerical model solution for destabilization damage to the coal-rock are displayed in Figs. 8, 9, 10 (unit: MPa).

The evolution of the stress field during the destabilization damage model follows a specific process. As depicted in Figs. 8a, 9a, 10a, the stress field diminishes and forms bands from top to bottom under axial loading, with a high-stress region generated at the top. With increasing axial stress, the stress field tends to distribute uniformly until a local unit stress imbalance and stress difference formation occur, indicating a tendency toward destabilization, as shown in Figs. 8b, 9b, 10b. Low-stress bands crossing along the yield surface are evident in Figs. 8c, 9c, 10c. In Figs. 8d, 9d, the low-stress band in the coal-rock monoliths initially appears at the top-middle position and then rapidly expands toward the bottom. As low-stress strips continue to form, the unit strain on the yield surface exceeds a predefined failure limit, leading to the formation of macroscopic cracks, and the stress field tends toward a disordered state. By comparing Figs. 8 and 9, the difference in mechanical properties results in higher peak stress levels in Fig. 9a–d than those in Fig. 8a–d. Additionally, the peak stress levels in Figs. 9(e) are lower than those in Fig. 8e during destabilizing damage. This finding aligns with the results of mechanical property tests, corresponding to the rapid stress decay observed after destabilization damage in rock samples^43^. It further confirms the validity of the model’s stress field evolution.

In addition to the previously mentioned commonality in stress field evolution, the complex destabilization damage process demonstrates its unique stress field evolution behavior. In Fig. 10b, it is evident that the stress in the “coal” section is considerably lower than that in the “rock” section, and a differential stress region is preferentially formed at the interface. Ongoing axial loading generates a high-stress area at the interface and a low-stress area at the bottom of the “coal” section, indicating a tendency for destabilization damage. In Fig. 10c, the distribution of stress field weakening makes the interface and the “coal” section bottom susceptible to high-stress differentials, leading to destabilization. In Fig. 10e, the complex undergoes irreversible deformation, resulting in numerous low-stress regions. A small amount of high-stress region still exists at the interface, forming a high-stress difference with the surrounding region, indicating a tendency for destabilization damage at the interface. Due to differences in the mechanical properties of coal and rock, the simultaneous existence of two regions of stress difference will lead to the destabilization damage tendency of both the interface and the bottom of the “coal” section^32^. In the simulation results, when the “coal” section is destabilized, the stress field at the interface is redistributed, weakening the destabilization tendency. Consequently, the macro crack expands from the bottom of the “coal” section toward the interface.

### Analysis of the evolution of destabilization damage

Through an examination of the stress field evolution behavior and IR radiation response signals during the destabilization damage process of coal, rock, and their complexes, the behavior of coal-rock matrix slip and dislocation damage facilitates the intensification of local molecular motion. This forms the fundamental basis for the phenomenon of infrared thermal image differentiation. Therefore, this study elucidates the distinctions in displacement vector and slip dislocation damage of coal, rock, and their complex through the displacement contour. Furthermore, it compares and analyzes the IR radiation response behavior to explore the destabilization damage evolution during the coal-rock complex. According to the IR radiation signal monitoring principle of the infrared camera, each pixel point in the measurement area corresponds to a two-dimensional temperature data matrix of the sample surface. The *p*-th frame temperature matrix is then represented as follows^46^.

11 $$f_{p} \left( {m,n} \right) = \left[ {\begin{array}{*{20}c} {f_{p} \left( {1,1} \right)} & {f_{p} \left( {1,2} \right)} & \cdots & {f_{p} \left( {1,L_{n} } \right)} \\ {f_{p} \left( {2,1} \right)} & {f_{p} \left( {2,2} \right)} & \cdots & {f_{p} \left( {1,L_{n} } \right)} \\ \vdots & \vdots & \ddots & \vdots \\ {f_{p} \left( {L_{m} ,1} \right)} & {f_{p} \left( {L_{m} ,2} \right)} & \cdots & {f_{p} \left( {L_{m} ,L_{n} } \right)} \\ \end{array} } \right]$$

where: *m* is the matrix row number, *n* is the matrix column number, *L*_*m*_ is the maximum number of matrix rows, and *L*_*n*_ is the maximum number of matrix columns.

**Figure 11:**
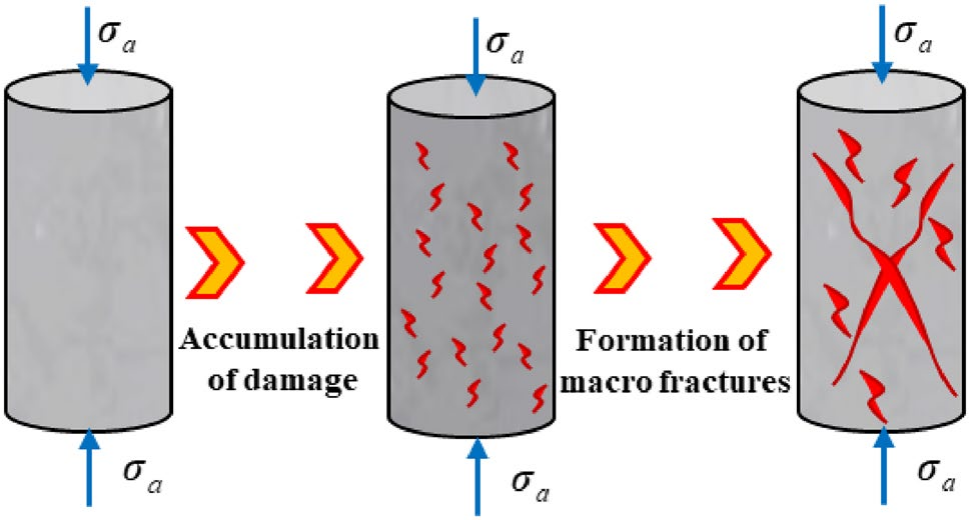
Damage evolution.

**Figure 12:**
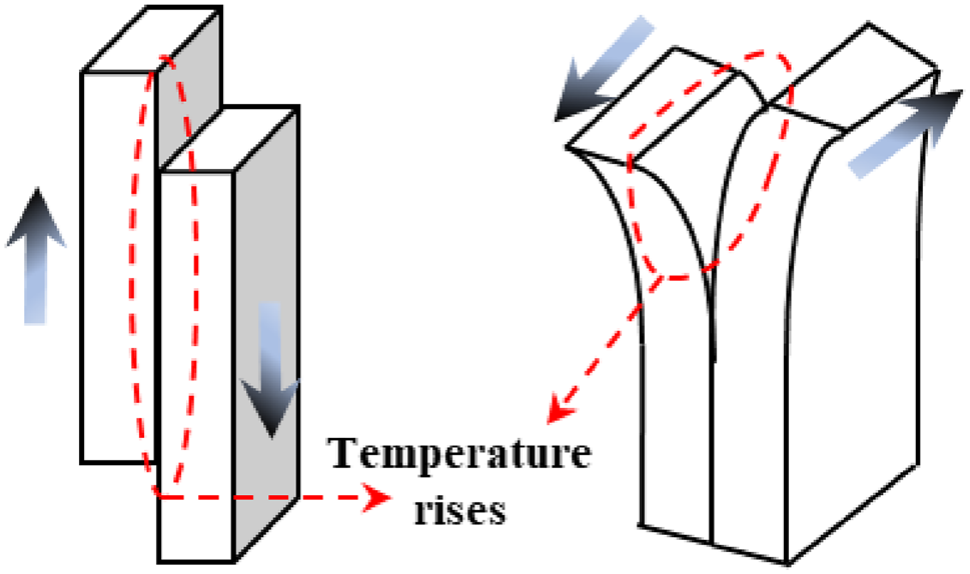
Temperature rise from shear damage.

The IR radiation signal during the destabilization damage process of the coal-rock is generally weak and prone to interference from background noise. Therefore, the differential radiation temperature method was chosen to process the infrared video and initiate the analysis^47^, as illustrated in Fig. 7. Throughout the destabilization damage process of the complex, the matrix of the coal-rock will persistently experience shear-tension damage under the influence of external forces. Upon the accumulation of a certain level of damage, macroscopic cracks will develop, as depicted in Fig. 11. The occurrence of shear damage leads to slip dislocation between the coal-rock matrices, resulting in the phenomenon of temperature rise and intensifying molecular movement, as illustrated in Fig. 12.

**Figure 13:**
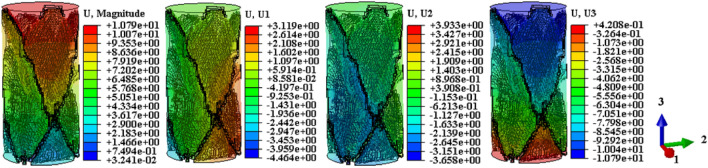
Coal monomer displacement cloud evolution.

**Figure 14:**
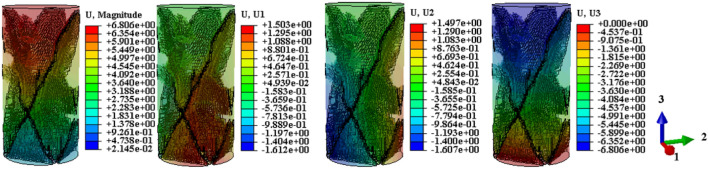
Rock monomer displacement cloud evolution.

**Figure 15:**
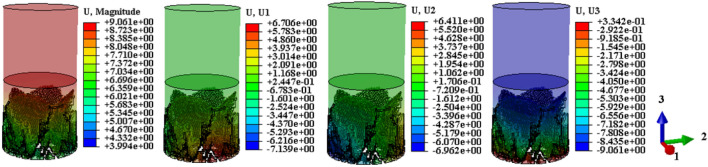
Complex monomer displacement cloud evolution.

Drawing upon the relationship between the temperature field and the damage evolution behavior of the sample, this analysis explores the displacement cloud and IR radiation response characterization of the destabilization damage process. The aim is to provide theoretical support for the study and prediction of the damage evolution behavior of the coal-rock during destabilization. Considering that shear damage is the primary cause of the anomalous increase in the local temperature field^12^, the displacement maps on U1, U2, and U3 in mutually perpendicular directions are proposed for selection here. This choice is conducive to the comparative analysis of shear damage between the coal and rock substrates. The displacement contours resulting from the calculated model destabilization damage are presented in Figs. 13, 14, 15 (unit: mm).

To compare displacement differences in various regions of coal, rock, and their complexes, incremental steps were selected, corresponding to instances where a significant number of model units failed, and macroscopic cracks emerged. The results are depicted in Figs. 13, 14, 15. Figures 13, 14, 15 reveal that cracks, formed by failed units, divide the model into distinct regions with significantly differing displacement contours. Analyzing misalignment, slippage, and shear damage that occurs at the cracks between regions is facilitated by comparing different color divisions. Upon comparing Figs. 13, 14, 15, it was observed that monoliths formed “X” cracks, whereas cracks in the complexes expanded from the coal bottom to the interface, forming extremely dense cracks. This observation aligns with the phenomenon that the IR thermal image of the “coal” section in the test exhibits obvious differentiation characteristics, and the damage degree has increased^38^. In monoliths, the tops and bottoms of the cracks are compressed in the direction of U3, resulting in the lateral displacements U1 and U2. In the complexes, lateral displacement is predominantly observed at the bottom of the “coal” section, and the displacement contours at the interface resemble those of the “rock” section. Combining the displacement contours with the analysis of stress field evolution in Fig. 10, two main reasons are identified for the phenomenon of concentrated lateral displacement. Firstly, the coal-rock matrix at the interface will undergo different degrees of deformation simultaneously, and this deformation will suppress the destabilization damage tendency of the coal matrix at the interface. Secondly, there is no low-stress zone at the interface; that is, the stress difference at the “coal” section bottom is higher than at the interface. Drawing on the above analysis, the anomalous high-temperature scattering-intensive phenomenon of the infrared radiation signal in the destabilization damage of the complex results from the local dislocation and slippage of the coal-rock, coupled with the frictional heat effect. This effect gives rise to the anomalous high-temperature banding depicted in Fig. 7e, f, primarily concentrated in the “coal” section and the interface. Ultimately, this leads to cracks at the bottom of the “coal” section extending towards the interface.

## Conclusions

- (1) The maximum UCS of the cast complex samples, consisting of similar materials in the tests, surpassed that of the coal samples but remained significantly lower than that of the rock samples. The axial strain during the final complete destruction exhibits a magnitude relationship as follows: CR (complex) > C (coal) > R (rock). The validity of the model was confirmed through a comparison of the plastic deformation contours and the instability damage patterns of the samples. The simulation reveals that a substantial number of cracks initiate and extend towards the interface at the bottom of the “coal” section, forming a wave-like deformation region at the interface. This is in contrast to the single extension pattern observed in the monoliths.
- (2) Both rock and coal samples exhibited anomalous high-temperature stripes, leading to damage along these anomalies and the subsequent formation of macro cracks. The rock samples exhibited more pronounced differences in the IR thermal images, with a more noticeable increase in surface temperature. Under complex loading conditions, the anomalous temperature scatters are notably denser in the ‘coal’ section compared to the ‘rock’ section. If the “rock” section displays a few anomalous high-temperature scatters, the “coal” section and interface have already developed anomalous high-temperature bands, indicating a tendency towards destabilization damage and the formation of macro-cracks.
- (3) The process of destabilizing monoliths exposes low-stress zones parallel to the yield surface that intersect, giving rise to a high-stress differential region leading to damage. In the course of complex destabilization damage, a red band of high stress emerges at the interface, subsequently creating a region of stress difference with the surrounding coal-rock body. Simultaneously, the bottom of the “coal” section, away from the interface, generates low-stress stripes extending towards the interface, forming a region of stress difference. Due to the influence of differing mechanical properties between coal and rock, the co-existence of two stress areas results in a destabilization tendency at both the interface and the bottom of the “coal” section in the complex. If the bottom of the “coal” section is destabilized first, the stress field at the interface is redistributed, mitigating the destabilization tendency.
- (4) The simulation of the destabilization damage process of the coal-rock complex reveals the formation of a significant number of “X”-shaped cracks at the bottom of the “coal” section. These cracks result from the slip displacement of numerous units, leading to the accumulation of damage and the intensification of local damage. This finding aligns with the experimental results. This aligns with the IR radiation response behavior observed during the destabilization damage process of the complexes. It is a major factor contributing to the concentration of frictional heat effect regions and the notable phenomenon of IR thermal image differentiation.

### Ethics approval

The manuscript has not been submitted to more than one journal for simultaneous consideration. The submitted work is original and has not been published elsewhere in any form or language (partially or in full). Results were presented clearly, honestly, and without fabrication, falsification or inappropriate data manipulation (including image based manipulation). The manuscript is our own work, and no data, text, or theories by others are presented unless cited.

## Data Availability

Data available on request from the corresponding author.
